# Y1 receptor deficiency in β-cells leads to increased adiposity and impaired glucose metabolism

**DOI:** 10.1038/s41598-018-30140-2

**Published:** 2018-09-03

**Authors:** Kim Loh, Yan-Chuan Shi, Mohammed Bensellam, Kailun Lee, D. Ross Laybutt, Herbert Herzog

**Affiliations:** 1 Neuroscience Division, Garvan Institute of Medical Research, St. Vincent’s Hospital, Sydney, 2010 Australia; 2 Diabetes and Metabolism Division, Garvan Institute of Medical Research, St. Vincent’s Hospital, Sydney, 2010 Australia; 3 Faculty of Medicine, UNSW Australia, Sydney, 2052 Australia; 4 St. Vincent’s Institute of Medical Research, Fitzroy, VIC 3065 Australia

## Abstract

Insulin secretion from pancreatic β-cells is critical for maintaining glucose homeostasis and deregulation of circulating insulin levels is associated with the development of metabolic diseases. While many factors have been implicated in the stimulation of insulin secretion, the mechanisms that subsequently reduce insulin secretion remain largely unexplored. Here we demonstrate that mice with β-cell specific ablation of the Y1 receptor exhibit significantly upregulated serum insulin levels associated with increased body weight and adiposity. Interestingly, when challenged with a high fat diet these β-cell specific Y1-deficient mice also develop hyperglycaemia and impaired glucose tolerance. This is most likely due to enhanced hepatic lipid synthesis, resulting in an increase of lipid accumulation in the liver. Together, our study demonstrates that Y1 receptor signaling negatively regulates insulin release, and pharmacological inhibition of Y1 receptor signalling for the treatment of non-insulin dependent diabetes should be taken into careful consideration.

## Introduction

Glucose homeostasis is a tightly regulated process coordinated by insulin and glucagon secretion. Located within the islets of Langerhans, pancreatic β-cells synthesize the hormone insulin, which is secreted primarily in response to elevated blood glucose concentrations^1,2^. In addition to this glucose dependent pathway, it is widely recognised that nutrients and hormones such as leptin, fatty acids, growth hormone and glucagon like peptide-1 (GLP-1) can regulate insulin release^3–5^. However, there is also evidence that shows insulin secretion can be regulated by the parasympathetic nervous system triggered by neuronal action within the arcuate nucleus of the hypothalamus^6–9^. Importantly, intracerebroventricularly administration of neuropeptide Y (NPY) significantly increases circulating insulin levels and this action can be blocked by vagotomy or treatment with muscarinic receptor blockers^7^. While there is now a wealth of information available that describes the mechanisms controlling insulin release, very little is known about the mechanisms to inhibit insulin secretion.

One of the known components of the counter-regulatory process is glucagon, which acts to prevent hypoglycemia by promoting glycogenolysis and increasing gluconeogenesis^10^. Additionally, adrenaline, noradrenaline and peptide YY (PYY) have also been implicated in the inhibition of insulin release^11–15^. PYY, a 36 amino acid peptide released from L-type cells of the gut and a member of the NPY family, is well recognised for its role to inhibit feeding and increase energy expenditure through activation of hypothalamic Y-receptors^13,16,17^. Importantly, however, PYY is also expressed in α-cells in the pancreatic islets where it is co-stored with glucagon in the secretory granules^18,19^, and its function in this tissue is less clear.

NPY as well as PYY act upon Y-receptors of which five receptors: Y1, Y2, Y4, Y5, and y6 are being cloned and characterized^20,21^. It is well established that central as well as peripheral actions of the NPY system are involved in the regulation of energy balance, with central pathways mostly promoting feeding via the activation of central Y1 receptors. In contrast, circulating PYY and its processed form PYY3–36, can both act on Y2 receptors located in brain areas with a semi-permeable blood brain barrier such as the arcuate nucleus (Arc) of the hypothalamus to mediate satiety^16,22,23^. In addition to central nervous system, Y1 receptors are also expressed in peripheral tissues including the liver, muscle, adipose tissue and pancreas, suggesting a potential role of the peripheral Y1 receptor signaling in regulating diverse physiological processes. In line with this, recently studies have shown that Y1 receptors in adipose tissues play a critical role in the regulation of adipogenesis and lipid oxidation^24^. Importantly, Y1 receptors are also expressed in the pancreatic islets, both in mice and humans^25–27^, however, the precise physiological role of Y1 receptors in the islets, in particular in the regulation of whole body glucose metabolism *in vivo* remains unknown.

Like all other Y-receptors, the Y1 receptor is a G-protein coupled receptor that preferentially associates with Gi/o G-protein and therefore acts in an inhibitory fashion, for example, reducing intracellular cyclic AMP (cAMP) levels in target cells^20^. Interestingly, it has been demonstrated that elevation of intracellular cAMP can promote insulin release and enhance pancreatic β-cell survival^28–32^. The presence of Y1 receptors in the islets raises a potential role for a direct inhibitory effect of cAMP and insulin release induced by Y1 receptor signalling. Indirect evidence from global Y1-deficient mice that exhibit hyperinsulinemia suggests a link between insulin secretion and Y1 receptor signaling in the islets *in vivo*^33^. However, the global deletion of Y1 receptors in mice leads to the development of obesity and impaired feeding^13,16,33^, and it remains therefore unclear as to whether hyperinsulinemia is the direct cause of Y1 receptor deficiency in the pancreas or the secondary effect of obesity.

To clarify the specific role of pancreatic Y1 receptors in islet function and glucose metabolism, we have generated pancreatic β-cell-specific Y1 receptor-deficient mice and examined the metabolic alterations that are caused by the lack of Y1 signaling in the pancreatic islets.

## Results

### Generation of the β-cell-specific Y1-deficient mice

**Figure 1:**
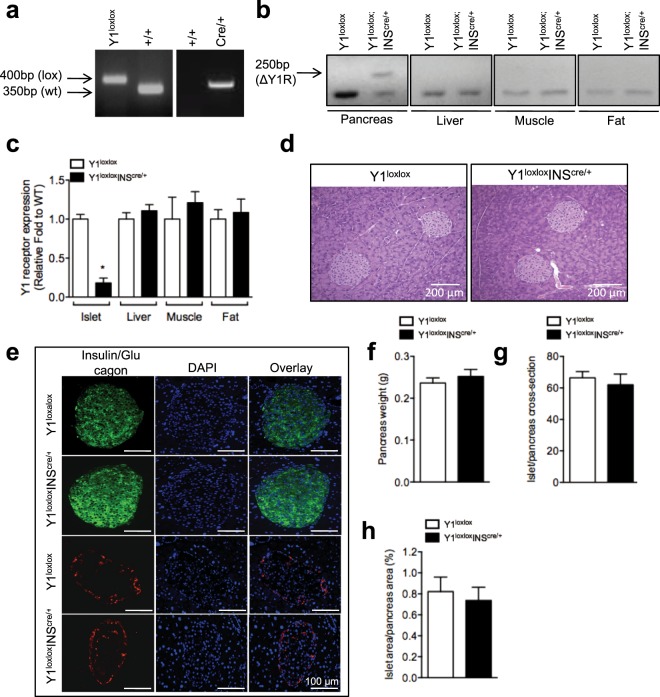
Generation of β-cell-specific Y1 receptor deficient mice. (**a**) Genotyping using PCR for determining WT, Y1^lox/lox^ and INS2^cre/+^ mice. (**b**) Assessment of recombination at the level of genomic DNA in pancreas, liver, muscle and fat of Y1^lox/lox^ and Y1^lox/lox^/INS2^cre/+^ mice. The 250 bp band corresponding to the deleted allele is only be found in pancreas. (**c**) Relative *Npy1r* mRNA expression determined in various tissues including the islets, liver muscle and adipose tissue by RT-PCR using RPL-19 as a housekeeping gene. (**d**) Pancreases from Y1^lox/lox^ and Y1^lox/lox^/INS2^cre/+^ mice were fixed in formalin and processed for immuno-stainings of hematoxylin and eosin (H&E) and (**e**) insulin (green), glucagon (red) and nuclear counterstained with DAPI (blue). (**f**) Pancreas weight of Y1^lox/lox^ and Y1^lox/lox^/INS2^cre/+^ mice. (**g**,**h**) Islet number and islet area were determined across three non-consecutive pancreatic sections per mouse and normalized to total pancreas section area. Data are means ± SEM of 4–10 mice per group. The cropped gel is used in the figure and full-length gel is presented in Supplementary Fig. 4.

To investigate the precise role of Y1 receptor signaling in islets, we generated a β-cell-specific Y1 receptor knockout model by crossing our conditional Y1^lox/lox^ with mice expressing the Cre recombinase gene under the control of the rat insulin-2 promoter (INS2^cre/+^) to generate (Y1^lox/lox^/INS2^cre/+^) mice. As shown in Fig. 1a,b, as proof of successful Y1 deletion, the recombinant product was only detected in genomic DNA samples isolated from pancreas tissue, but not liver or any other peripheral tissues of Y1^lox/lox^/INS2^cre/+^ mice (Fig. 1a,b). In addition, Y1 receptor expression was also assessed by quantitative RT-PCR, showing unaltered levels of Y1 receptor mRNA in the liver but a clear deficiency in expression in islets isolated from Y1^lox/lox^/INS2^cre/+^ mice (Fig. 1c). No alteration in Y1 receptor expression was noted in other peripheral tissues such as muscle and fat, further confirming that the specificity of the deletion is limited to islets (Fig. 1c). However, since it has been reported that INS2-cre expression is also found in the brain, we performed radio-ligand binding experiments on hypothalamic slices derived from control and Y1^lox/lox^/INS2^cre/+^ mice using the Y1 receptor preferring radiolabeled ligand, ^[Leu31,Pro34]^NPY. Results from this experiment clearly show that both control and Y1^lox/lox^/INS2^cre/+^ mice have similar total-specific binding counts (Supplementary Fig. 1a). In addition, hypothalamic Y1 receptor expression were similar in control and Y1^lox/lox^/INS2^cre/+^ mice as assessed by quantitative RT-PCR (Supplementary Fig. 1b), demonstrating that Y1 receptor expression in the hypothalamus is unaffected by INS2-cre expression.

### Lack of  Y1 receptors in β-cells does not alter islet morphology

To understand the underlying mechanisms of enhanced insulin secretion in Y1^lox/lox^/INS2^cre/+^ mice, we first evaluated whether lack of Y1 receptor signaling in pancreatic islets affects morphology and β-cell growth. We performed immunohistochemical analyses of pancreas sections using hematoxylin and eosin (H&E) staining as well as immunofluroscence staining for insulin and glucagon in control and Y1^lox/lox^/INS2^cre/+^ mice. Results from this revealed no obvious differences in islet morphology between these two groups (Fig. 1d,e). Consistent with this, pancreas weight, the number of islets and the average size of the islets per pancreas section were similar in control and Y1^lox/lox^/INS2^cre/+^ mice (Fig. 1f−h).

### Lack of  Y1 receptor signaling in β-cells results in increased body weight and adiposity

**Figure 2:**
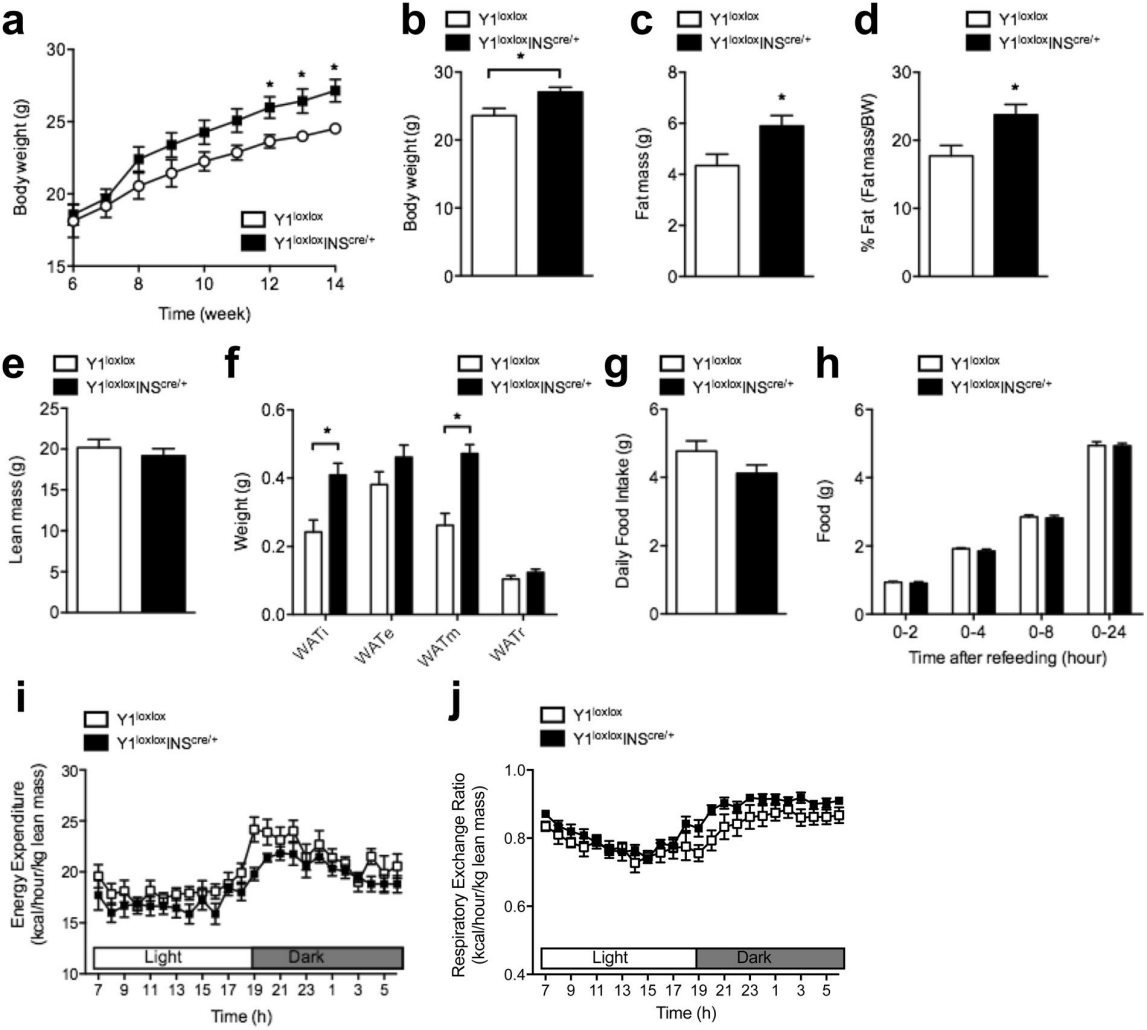
Increased body weight and adiposity in β-cell-specific Y1 receptor deficient mice. (**a**) Body weight curve of Y1^lox/lox^ and Y1^lox/lox^/INS2^cre/+^ mice. (**b**) Body weight at cull. (**c**−**e**) Absolute and normalised body composition (lean and fat mass) determined by DXA scan for 12–14-week-old Y1^lox/lox^ and Y1^lox/lox^/INS2^cre/+^ mice. (**f**) Absolute weights of adipose tissues from the inguinal (WATi), epidydimal (WATe), mesenteric (WATm) and retroperitoneal (WATr) depots at cull of chow fed Y1^lox/lox^ and Y1^lox/lox^/INS2^cre/+^ mice. (**g**,**h**) Daily and 24-hour fasting-induced food intake of Y1^lox/lox^ and Y1^lox/lox^/INS2^cre/+^ mice. (**i**,**j**) Light and dark cycle energy expenditure (normalised to total lean mass) and respiratory exchange ratio were determined in metabolic chambers during indirect calorimetry studies in 14–16-week-old Y1^lox/lox^ and Y1^lox/lox^/INS2^cre/+^ mice. Data are means ± SEM of 8–10 mice per group.

At 6–8 weeks of age, no differences in body weight (Fig. 2a) and body length (Supplementary Fig. 2a) were noted between control and Y1^lox/lox^/INS2^cre/+^ male mice, indicating that the deletion of Y1 receptor in the islets had no impact on early growth and development. Body composition and glucose homeostasis parameters were comparable between control and Y1^lox/lox^/INS2^cre/+^ (Supplementary Fig. 2b−h). Interestingly, however, when monitored over a longer period the body weight of Y1^lox/lox^/INS2^cre/+^ mice significantly increased compared to that of control littermates (Fig. 2a,b). The observed increase in body weight in the Y1^lox/lox^/INS2^cre/+^ mice was mostly due to an increase in fat mass as determined by whole-body Dual-energy X-ray absorptiometry (DXA) (Fig. 2c,d). By contrast, lean mass was similar in both Y1^lox/lox^/INS2^cre/+^ and control mice (Fig. 2e). Consistent with the DXA results, the absolute weights of dissected white adipose tissues were significantly elevated in 14-week-old male Y1^lox/lox^/INS2^cre/+^ mice with the inguinal (WATi) and mesenteric (WATm) depots contributing the most (Fig. 2f). No differences in body weight and body composition were evident in INS2^cre/+^ and INS2^+/+^ controls (Supplementary Fig. 3a−f), consistent with the difference in body weight being due to islet-specific Y1 receptor deficiency rather than ectopic effects of the INS2-cre transgene.

In addition, our results show that both spontaneous and fasting-induced food intake were similar between control and Y1^lox/lox^/INS2^cre/+^ mice (Fig. 2g,h), also further confirming that central Y1 receptors, which are acting stimulatory for food intake, are not affected by this insulin gene promoter driven Cre-expression. Results obtained from indirect calorimetric experiments suggest that energy expenditure and respiratory exchange ratio (RER; a measure of fat and carbohydrate utilization) were unaltered between Y1^lox/lox^/INS2^cre/+^ and control mice (Fig. 2i,j).

### Y1 receptor signaling in β-cells controls insulin levels

**Figure 3:**
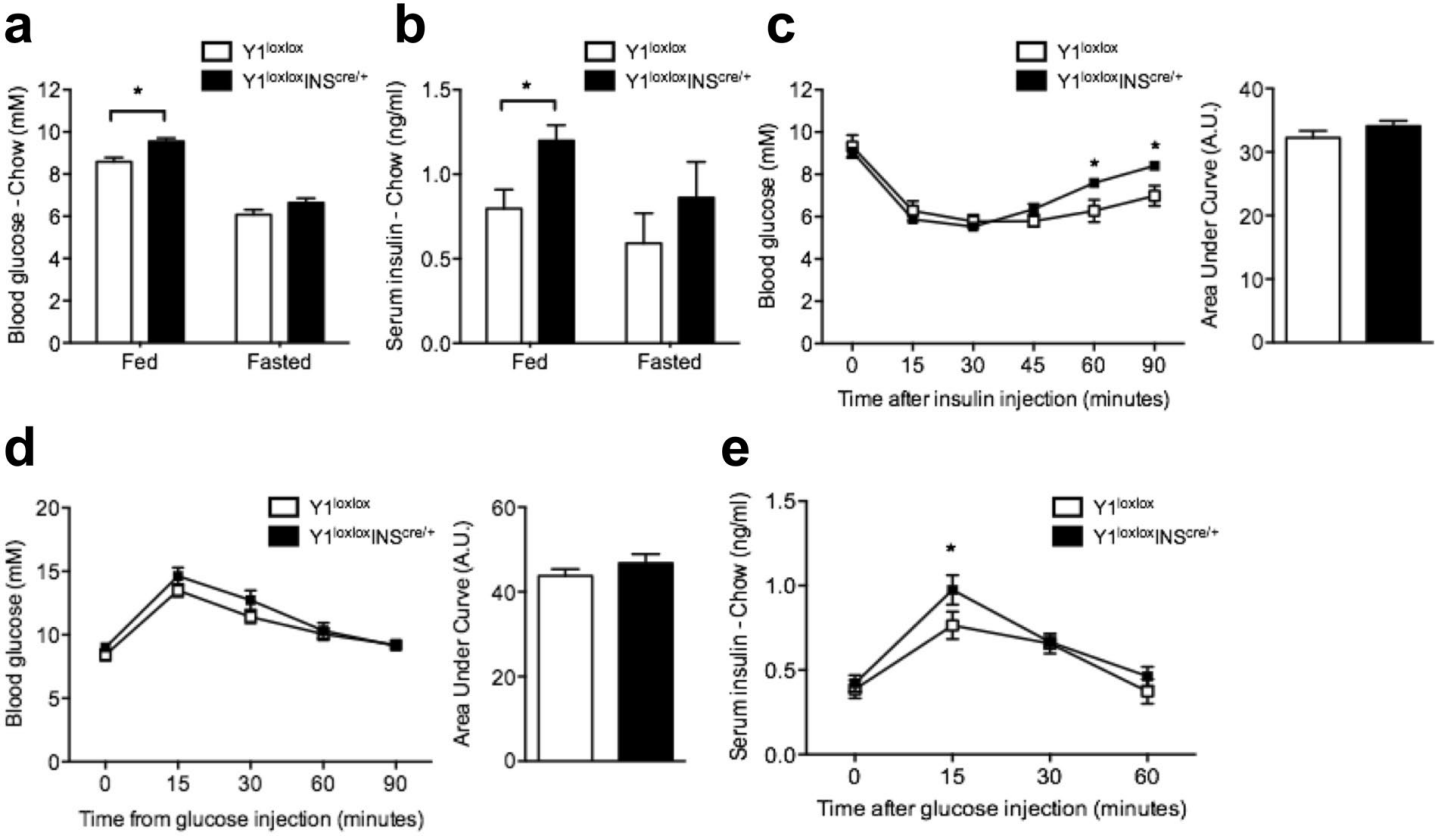
Enhanced circulating insulin levels and glucose-induced insulin secretion in β-cell-specific Y1 receptor deficient mice. (**a**,**b**) Non-fasted and fasted blood glucose and serum insulin levels in chow fed 12–14-week-old Y1^lox/lox^ and Y1^lox/lox^/INS2^cre/+^ mice. (**c**) Insulin tolerance test of chow fed Y1^lox/lox^ and Y1^lox/lox^/INS2^cre/+^ mice. Results also expressed as area under the curve. (**d**) Glucose tolerance test of chow fed Y1^lox/lox^ and Y1^lox/lox^/INS2^cre/+^ mice. Results also expressed as area under the curve. (**e**) Corresponding insulin levels from the GTT and resulting area under the curve. Results are means ± SEM of 8–10 mice per group.

To assess the effect of islet Y1 receptor deficiency on the overall regulation of glucose homeostasis, we first measured fed and fasted blood glucose and insulin levels in chow control and Y1^lox/lox^/INS2^cre/+^ mice. Importantly, our results show that blood glucose levels in the fed state were significantly elevated in chow Y1^lox/lox^/INS2^cre/+^ mice (Fig. 3a). Interestingly, Y1^lox/lox^/INS2^cre/+^ mice also exhibited a significant increase in circulating serum insulin levels in the fed state when compared to control mice (Fig. 3b), indicating that selective Y1 receptor deletion in islets contributes to the development of hyperinsulinemia. Although there was a tendency towards increased basal fasting insulin levels in Y1^lox/lox^/INS2^cre/+^ mice, this was not statistically significant (Fig. 3b). Whole-body insulin action evaluated by insulin tolerance tests showed no significant differences between the two genotypes; however insulin responses in control mice were more prolonged at 60 and 90 minutes (Fig. 3c).

Next, we investigated the impact of islet-specific Y1 receptor deficiency on insulin secretion in response to intraperitoneal (i.p.) administration of glucose. Both chow fed control and Y1^lox/lox^/INS2^cre/+^ mice exhibited similar glucose levels at all time points after glucose administration (Fig. 3d). As expected, 15 mins after glucose administration, serum insulin levels were elevated in both control and Y1^lox/lox^/INS2^cre/+^ mice, however the elevation was significantly more pronounced in the Y1^lox/lox^/INS2^cre/+^ compared to control mice (Fig. 3e). To ensure that this observed glucose intolerance phenotype was not due to non-specific actions of the presence of INS2-cre recombinase expression, we compared INS2^cre/+^, Y1^lox/+^ and Y1^lox/+^/INS2^cre/+^ and found no difference in blood glucose levels, insulin sensitivity and glucose tolerance between these groups (Supplementary Fig. 3g−i). These results demonstrate that the impairment in glucose homeostasis in Y1^lox/lox^/INS2^cre/+^ mice is a direct consequence of the lack of Y1 receptor signaling in β-cells and confirms a critical role of this Y-receptor in the regulation of insulin secretion and glucose metabolism.

### Beta-cell-specific Y1-deficient mice show exacerbated diet-induced obesity

**Figure 4:**
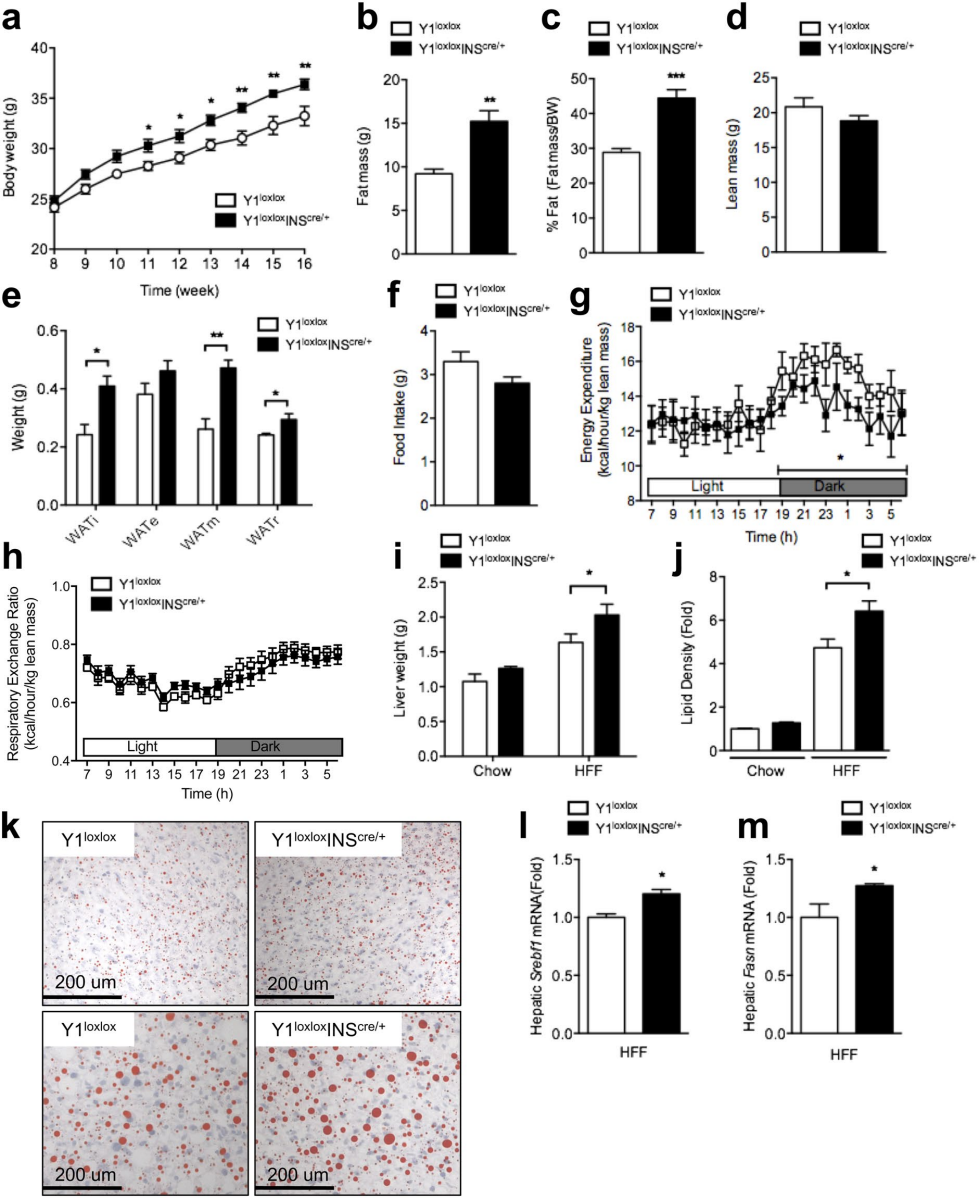
Mice lacking Y1 receptor in β-cells showed exacerbated diet-induced obesity. (**a**) Body weight curve of Y1^lox/lox^ and Y1^lox/lox^/INS2^cre/+^ mice fed on a high fat diet for 8 weeks. (**b**−**d**) Absolute and normalised body composition (lean and fat mass) determined by DXA scan. (**e**) Weight of white adipose tissues from the inguinal (WATi), epidydimal (WATe), mesenteric (WATm) and retroperitoneal (WATr) depots in high fat fed Y1^lox/lox^ and Y1^lox/lox^/INS2^cre/+^ mice. (**f**−**h**) Food intake, energy expenditure and respiratory exchange ratio in high fat fed Y1^lox/lox^ and Y1^lox/lox^/INS2^cre/+^ mice. (**i**) Liver weight and (**j**) liver morphology were determined by the quantity of oil-red-o staining from chow- and high fat fed Y1^lox/lox^ and Y1^lox/lox^/INS2^cre/+^ mice. (**k**) Representative oil-red-o stained liver sections from Y1^lox/lox^ and Y1^lox/lox^/INS2^cre/+^ mice. (**l**,**m**) Quantitative RT-PCR determining hepatic lipogenic gene *Srebf1* and *Fasn* from livers from high fat fed Y1^lox/lox^ and Y1^lox/lox^/INS2^cre/+^ mice using *Rlp19* as a housekeeping gene. Data are means ± SEM of 8–10 mice per group.

Although it has been shown that chow fed Y1^lox/lox^/INS2^cre/+^ mice display an increase in body weight and adiposity, it remains unclear whether β-cell specific Y1 receptor deficiency can prevent or further exacerbate the development of diet-induced obesity. To assess this, 8 week-old control and Y1^lox/lox^/INS2^cre/+^ mice were fed on a high fat diet for 8 weeks. Surprisingly, Y1^lox/lox^/INS2^cre/+^ mice are highly responsive to high fat feeding with a significantly increased body weight gain compared to control littermates under these conditions (Fig. 4a). Consistent with the effect in mice on a normal chow diet, high fat fed Y1^lox/lox^/INS2^cre/+^ mice displayed significantly greater fat mass and relative adiposity, while lean mass was unaltered (Fig. 4b−d). In line with this, Y1^lox/lox^/INS2^cre/+^ mice exhibited a significant elevation in weights of adipose tissue depots after 8 weeks of high fat feeding (Fig. 4e). Similar to the chow fed mice, Y1^lox/lox^/INS2^cre/+^ mice fed on high fat diet displayed no difference in energy intake (Fig. 4f). While respiratory exchange ratio was comparable, the increase in body weight and adiposity in high fat-fed Y1^lox/lox^/INS2^cre/+^ mice was accompanied by a significant decrease in energy expenditure (Fig. 4g,h), partially explaining the development of diet-induced obesity.

Since obesity is associated with ectopic lipid deposition in various tissues including the liver, we investigated hepatic lipid content in Y1^lox/lox^/INS2^cre/+^ mice. Although no differences in liver weights were noted in chow fed control and Y1^lox/lox^/INS2^cre/+^ mice, the liver weights in high fat-fed obese Y1^lox/lox^/INS2^cre/+^ mice were significantly elevated (Fig. 4i). This was associated with a significant increase in hepatic lipid accumulation in the livers of these mice as determined by oil-red-o staining (Fig. 4j,k). Interestingly, the increased hepatic lipid accumulation was associated with enhanced lipogenic gene *Srebf1* and *Fasn* mRNA levels (Fig. 4l,m), consistent with the enhanced adiposity exhibited by Y1^lox/lox^/INS2^cre/+^ mice.

### Impaired glucose homeostasis in HFF β-cell specific Y1-deficient mice

**Figure 5:**
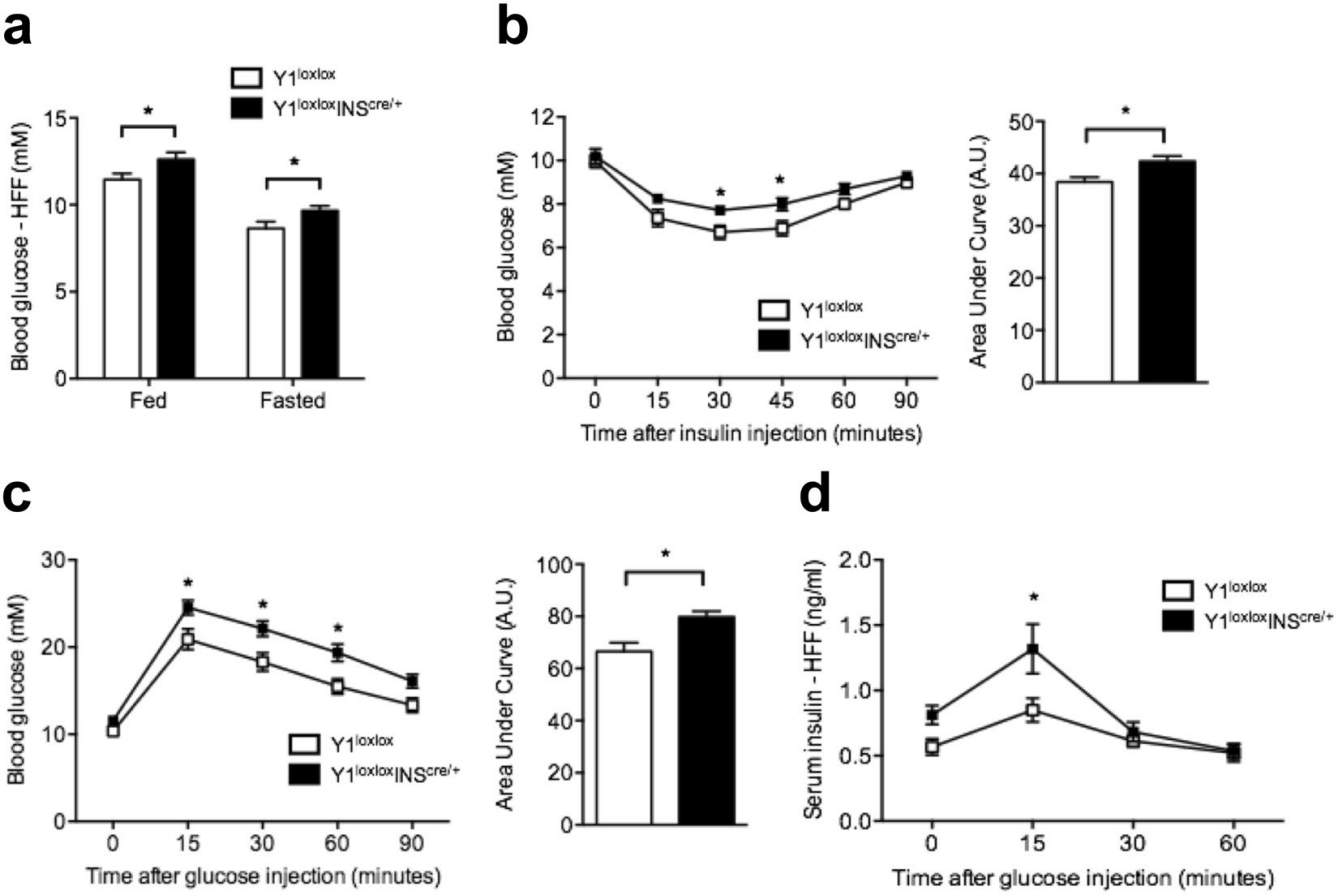
Impaired glucose homeostasis in β-cell-specific Y1 receptor deficient mice. (**a**) Blood glucose levels of non-fasted and fasted Y1^lox/lox^ and Y1^lox/lox^/INS2^cre/+^ mice fed on a high fat diet for 8 weeks. (**b**,**c**) Insulin and glucose tolerance tests of high fat fed Y1^lox/lox^ and Y1^lox/lox^/INS2^cre/+^ mice. Results are also expressed as area under the curve. (**d**) Corresponding insulin levels from the GTT. Results are means ± SEM of 8–10 mice per group.

We previously showed that HFF Y1^lox/lox^/INS2^cre/+^ mice exhibited a significant increase in circulating serum insulin levels in the fed state when compared to control mice^27^, here we also found that fed and fasted blood glucose levels were significantly elevated in HFF Y1^lox/lox^/INS2^cre/+^ mice (Fig. 5a). Furthermore, reduced insulin responsiveness was observed in HFF Y1^lox/lox^/INS2^cre/+^ mice, which coincided with significantly reduced glucose tolerance as determined by insulin and glucose tolerance tests, respectively (Fig. 5b,c). Interestingly, despite impaired glucose homeostasis, HFF Y1^lox/lox^/INS2^cre/+^ mice showed significantly higher glucose-stimulated insulin release, 15 mins after glucose administration, compared to control mice (Fig. 5d), suggesting that the ability to secrete insulin in response to glucose in Y1^lox/lox^/INS2^cre/+^ remains enhanced under high fat feeding state.

## Discussion

In this study, we demonstrate that Y1 receptor signaling in the islets is a critical regulator of insulin secretion since lack of Y1 receptors specifically in β-cells leads to a hyper-insulinemic state. Furthermore, Y1^lox/lox^/INS2^cre/+^ mice exhibit significantly increased blood glucose levels and developed glucose intolerance, while glucose-stimulated insulin secretion is significantly elevated *in vivo*. This altered insulin secretion then leads to an obese phenotype in these mice that is worsened by exposure to a high fat diet.

From a mechanistic point this is not due to altered islet morphology in the absence of Y1 signaling, but is caused by an increase in expression of specific key molecules that control insulin release. It is well established that glucose is the major regulator of insulin secretion by pancreatic β-cells. This process is initiated by the transportation of glucose via GLUT2 into β-cells. Subsequently, intracellular glucose is phosphorylated by glucokinase and metabolized by glycolysis and the Krebs cycle to generate ATP. The increase in cytosolic ATP/ADP ratio leads to the closure of ATP-gated potassium channels resulting in plasma membrane depolarization. This in turn leads to the opening of the voltage-gated calcium channels, increases intracellular calcium concentration and triggers insulin granule exocytosis^34–36^. This is supported by our published results showing that lack of Y1 receptor signaling in islet leads to significant changes in the expression of several genes involved in glucose metabolism that play critical roles in the insulin secretory pathway including glucokinase, pyruvate carboxylase and ATP citrate lyase^27^. Indeed, glucose hypersensitivity in rat islets has been shown to correlate with increased glucokinase activity and expression^37^. On the other hand, glucokinase mutations lead to impaired insulin secretion whereas glucokinase activators have been shown to enhance glucose-stimulated insulin secretion^38–41^. Interestingly, we also found a marked upregulation of the anaplerotic gene, pyruvate carboxylase. These results are in agreement with previous studies showing that overexpression of pyruvate carboxylase increased glucose-stimulated insulin secretion while its inhibition had the opposite effect^42–45^.

Recent studies have shown that cAMP is important for potentiating glucose stimulated insulin secretion^28–32^. For instance, studies have shown that glucagon-like peptide 1 (GLP1), activates cAMP dependant pathway to enhance glucose-induced insulin secretion and that reduced cAMP expression with antisense or inhibition of cAMP pathway abolishes these effects^28,46–49^. This indicates that cAMP may also serve as an intracellular signaling molecule in the insulin secretory pathway. Like all Y-receptors, the Y1 receptor is a G-protein coupled receptor that couples to adenylyl cyclase through association with Gi/o GTP-binding-protein and therefore reduces intracellular cyclic AMP production^20^. Our recent published results confirm that lack of Y1 receptor signaling in islets leads to an up-regulation of cAMP levels that are critical in altering gene expression of glucose metabolic enzymes and thereby enhancing insulin secretion^27^.

The NPY system is known to play a critical role in a variety of aspects of energy homeostasis^20,21^. Centrally, NPY is upregulated in the arcuate nucleus under conditions of negative energy balance, thereby inducing an increase in food intake and a simultaneous reduction in energy expenditure by down-regulating BAT activity^50^. Furthermore, it has been shown that elevated central NPY levels alter energy utilization by promoting fatty acid storage over fatty acid oxidation^51^. Importantly, central NPY is also known to act upon the autonomous nervous system and amongst other consequences of that leads to an increase in pancreatic insulin secretion mediated locally via muscarinic pathways^7^. On the other hand, Y1 receptors are also expressed directly on β-cells both in mouse and in human tissue suggesting a direct role of NPY or more likely one of its family members peptide YY (PYY) or pancreatic polypeptide (PP) to control functions of these cells.

In contrast to NPY, PYY and PP are produced locally in the islet in specific α-cells^18,19^ and PP cells^52,53^ respectively. Importantly, PYY and PP levels are known to increase in response to food intake and they can act as satiety factors on specific Y-receptors in the brain^13,16,17,54,55^. A local increase in PYY or PP in the pancreatic islet would therefore also be very timely to counter-regulate the increase of insulin secretion postprandial. Interestingly, PYY-deficient mice exhibit enhanced insulin secretion in response to glucose administration, and develop chronic hyperinsulinemia, while PYY over-expressing mice show the opposite phenotype^13,16^. This is further confirmed by the findings that exogenous application of PYY decreases glucose stimulated insulin secretion from rat and mouse islets^14,15^. Considering the close proximity of the PYY and PP expressing α-cells to the pancreatic β-cells, it is conceivable that PYY and/or PP via a paracrine action are involved in the counter-regulatory process of insulin secretion by activating Y1 receptor signaling.

Recent studies have shown that raised pancreatic PYY levels in diabetic rodents following Roux-en-Y gastric bypass (RYGB) surgery leads to significantly improved β-cell secretory function and restored islet morphology^56,57^. In addition to the regulation of β-cell function, PYY has been shown to promote β-cell proliferation and survival and prevent β-cell apoptosis^58–60^. This is supported by studies showing that adult onset ablation of PYY expressing cells leads the disruption of islet morphology and significantly decreased insulin content^59^. Similarly, the increase in β-cell proliferation by PYY was also observed in immortalised human 1.1B4 and rodent BRIN-BD11 β-cell lines^60^. Our results showing no difference in islet morphology such as β-cell number or β-cell mass in β-cell specific Y1 deficient mice suggests that the regulation of β-cell survival and proliferation is unlikely controlled via the Y1 receptor signalling but may involve other Y receptors such as the Y2 receptor.

Although activation of Y1 receptor likely reduces insulin secretion from β-cells, which could be beneficial in type 2 diabetes^61^, this is unlikely to be the main factor to impaired glucose homeostasis in high fat fed Y1^lox/lox^/INS2^cre/+^ mice. On the other hand, the role of insulin in lipogenesis has been extensively studied using several animal models of obesity^62,63^. Results obtained from our Y1^lox/lox^/INS2^cre/+^ mice are in agreement with a recent study showing that circulating hyperinsulinemia could drive diet-induced obesity in the absence of insulin resistance and glucose intolerance^64^. In addition to regulating glucose metabolism, insulin has also been shown to act in the liver to regulate lipid metabolism and inhibit lipolysis in white adipose tissue. Insulin activates *Srebp1* transcription, which in turn promotes the transcription of genes involved in fatty acid and triglyceride synthesis, including fatty acid synthase (*Fasn*), thus markedly increasing hepatic lipid accumulation^62,63,65^. In addition to increased serum insulin levels, high fat fed Y1^lox/lox^/INS2^cre/+^ mice have significantly increased liver weight and this was associated with increased hepatic lipid accumulation. The increase in lipid accumulation was accompanied by significantly increased hepatic *Srebp1* and *Fasn* expression, suggesting that lipogenesis is enhanced in the Y1^lox/lox^/INS2^cre/+^ mice. Previous studies have demonstrated that the deposition of fat in non-fat tissue such as liver could contribute to the development of insulin resistance and metabolic abnormalities^62,63^. Therefore, the increased lipogenesis as a consequence of elevated insulin levels may, at least in part, contribute to the development of obesity and impaired glucose homeostasis in Y1^lox/lox^/INS2^cre/+^ mice.

In summary, our results provide new evidence for the Y1 receptor acting in a counter-regulatory fashion in insulin release *in vivo* and suggest that impaired Y1 receptor signaling in the pancreas may play a causal role in the development of metabolic diseases such as obesity. While chronically increased levels of insulin may have negative side effects, in particular when combined with a high fat diet, under conditions of minimal or total lack of insulin supply, the phenotype of Y1 deficient islets might be an advantage. Importantly, our studies indicate that short-term inhibition of Y1 receptors in β-cells via pharmacological intervention may be beneficial under a condition where elevated insulin is required, such as in type 1 diabetes.

## Materials and Methods

### Mice

All animal care and experiments were approved by the Garvan/St. Vincent’s Animal Ethics Committee. All animal studies were conducted following the guidelines of the institutional animal ethics committee and the experiments were carried out in accordance with the approved guidelines. Y1^lox/lox^ mice were generated as previously described^66^ and crossed with mice expressing the Cre recombinase gene under the control of the rat insulin-2 promoter Tg(Ins2-cre)25Mgn/J (INS2^cre/+^)^67^ to generate Y1^lox/lox^/INS2^cre/+^ mice. Age- and sex-matched mice on a mixed C57BL/6–129SvJ background were used for all experiments. Mice were housed under a controlled temperature of 22 °C and a 12-hour light cycle (lights on from 07:00 to 19:00 hours) with *ad libitum* access to water and a standard chow diet (6% calories from fat, 21% calories from protein, 71% calories from carbohydrate, 2.6 kcal/g, Specialty Feeds, Australia) or fed a high fat diet (43% kilojoules from fat, 17% kilojoules from protein, 40% kilojoules from carbohydrate, 4.8 kcal/g, Specialty Feeds, Australia) as indicated.

### Metabolic measurements and tissue collection

Weekly body weight was determined from 6 weeks of age onwards, unless otherwise stated. Food intake was examined in chow- and high fat-fed mice under normal and fasting conditions as described previously^68^. Whole-body lean and fat masses were determined by DXA (Lunar Piximus II mouse densitometer; GE Healthcare, UK). Mice were scarified by cervical dislocation and trunk blood was collected, centrifuged and serum was obtained for further analysis. Tissues including brain, liver, muscle and white adipose tissue from the inguinal, epididymal, retroperitoneal and mesenteric deposits were weighed and stored at −20 °C.

### RNA extraction and quantitative RTPCR

Different tissues including liver, pancreatic islets were dissected and immediately frozen in liquid N_2_, and RNA was extracted using Trizol Reagent (Sigma, St. Louis, MO). Isolated mRNA was reverse transcribed into cDNA using Superscript III First-Strand Synthesis System (Invitrogen, Australia) and processed for quantitative real-time PCR (qPCR) with the Light-Cycler 480 Real-Time PCR system (Roche, Switzerland) for *Srebp1c* and *Fasn* and normalized with a housekeeping gene Ribosomal protein L19 (*Rpl-19)* (5′-CTCGTTGCCGGAAAAACA-3′ and 5′-TCATCCAGGTCACCTTCTCA-3′). *Npy1r* (Y1 receptor) gene (5′-CACAGGCTGTCTTACACG-3′ and 5′-GCGAATGTATATCTTGAAGTAG-3′).

### Glucose and insulin tolerance tests

Glucose and insulin tolerance tests were performed on 6 h or 4 h fasted mice that were administered i.p. with glucose (1 mg/g body weight) or insulin (0.75 mU/g body weight) respectively. Blood glucose levels were assessed at 0, 15, 30, 60 and 90 minutes after glucose administration using a Accu-chek® Go glucometer (Roche, Dee Why, Australia). Serum from mice administered with glucose was collected and stored for subsequent insulin assay as described previously. Fed and fasted serum insulin levels were measured using a Rat/Mouse Insulin RIA kits (Linco Research, St. Charles, MO, USA).

### Oil-Red-O and pancreatic histology

Frozen livers sections (6 µm) were cut on a cryostat, mounted on slides, fixed for 30 minutes in 4% PFA and processed for Oil-red-O staining as described previously^68^. To quantify lipid infiltration in the liver, Oil Red O stained sections were assessed on a grey scale for stained pixels within a defined frame using a light microscope (Leica, Heerbrugg, Switzerland) at 40x magnification. For islet morphology assay, insulin, glucagon, DAPI, hematoxylin and eosin (H&E) immunostaining was performed on paraformaldehyde-fixed, paraffin-embedded pancreatic sections, as described previously^58^. Briefly, whole pancreas were fixed in 4% PBS-buffered paraformaldehyde overnight at 4 °C before being processed and embedded in paraffin. Sections were cut at 5 μm, deparaffinized, rehydrated, and incubated with a Target Retrieval Solution (DAKO Corp) in a pressure cooker. Slides were then washed in distilled water and incubated with 5% goat serum in PBS containing 1% BSA for 1 hour at room temperature. Subsequently sections were incubated overnight at 4 °C in hydration chambers with the respective primary antibody. Slides were then washed in PBS and incubated with the respective secondary antibody for 1 hour at room temperature. The slides were washed in PBS and cover slipped with ProLong Gold Antifade mounting medium (Thermo Fisher Scientific, Inc). Entire sections of the pancreas were scanned and imaged using a Leica DM6000 microscope with LAS Power Mosaic. Total number of islets, islet areas and total pancreatic areas of sections were quantified using Image J software. Three sections separated by at least 150 μm were used for each mouse. A randomly select 10 islets per sections were counted to determine islet area/pancreas area. n = 4 mice per group.

### Indirect calorimetry

In order to determine energy expenditure, respiratory exchange ratio (RER) and physical activity, 14–16 weeks old mice fed on a normal chow diet, or 16-week old mice that had been maintained on the high fat diet for 8 weeks, were transferred to individual cages (20.1 × 10.1 × 12.7 cm) in an eight chamber open-circuit calorimeter (Oxymax Series; Columbus Instruments, Columbus, OH, USA). Temperature was maintained at 22 °C, with an airflow of 0.6 L/min. Mice were acclimatized to the cages for 24 hours before beginning 24-hour recording of oxygen consumption (VO_2_) and carbon dioxide production (VCO_2_). RER was calculated as VCO_2_ ÷ VO_2_. Energy expenditure (kilocalories of heat produced) was calculated as calorific value (CV) × VO_2_, where CV = 3.815 + 1.232 x RER as previously published (Melgar, 2007 #4915). Lean mass-normalized energy expenditure was calculated as energy expenditure divided by lean mass, which was determined immediately following the completion of indirect calorimetry using DXA as described above. Physical activity was also measured, using an OPTO-M3 sensor system (Columbus Instruments, Columbus, OH, USA), whereby ambulatory counts were a record of consecutive adjacent photo beam breaks in the horizontal space. Data for the 24-h monitoring period was presented as hourly averages for VO_2_, VCO_2_, RER, and energy expenditure as well as hourly summation for ambulatory activities. The calorimeter was calibrated before each use using highly pure primary gas standards (O_2_ and CO_2_).

### Radioimmunoassay and ELISA kits

Serum insulin levels were measured using the following kits: a Rat/Mouse Insulin ELISA kit (Linco Research, St. Charles, MO, USA) in accordance with the manufacturers’ specifications.

### Statistical Analyses

All data are expressed as mean ± SEM. A two-tailed student’s t-test was used to test difference between 2 groups of mice. Differences among groups of mice were assessed by two-way ANOVA or repeated-measures ANOVA. Bonferroni post-hoc tests were performed to identify differences among means. Statistical analyses were assessed using Prism software (GraphPad Software, Inc, LaJolla, CA, USA). Differences were regarded as statistically significant if *p value < 0.05; **p value < 0.01; ***p value < 0.001.

### Data availability

The datasets generated during and/or analysed during the current study are available from the corresponding author on reasonable request.

## Electronic supplementary material

Supplementary Information
