# Supplementary material for: Y1 receptor deficiency in β-cells leads to increased adiposity and impaired glucose metabolism
Source: Sci Rep. 2018 Sep 3;8:11835. doi: 10.1038/s41598-018-30140-2 (PMC6120893; doi:10.1038/s41598-018-30140-2)
Supplement: Supplementary file 1 — Supplementary Information [file 41598_2018_30140_MOESM1_ESM.pdf]

SUPPLEMENTARY DATA

Y1 receptor deficiency in β-cells leads to increased adiposity and impaired glucose metabolism.

Kim Loh[1,3,4*], Yan-Chuan Shi[1,3], Mohammed Bensellam[2], Kai Lun Lee[1,2], D. Ross Laybutt[2] and Herbert Herzog[1,3*]

[1]Neuroscience Division, Garvan Institute of Medical Research, St Vincent's Hospital, Sydney, 2010, Australia.

[2]Diabetes Division, Garvan Institute of Medical Research, St Vincent's Hospital, Sydney, 2010, Australia.

[3]Faculty of Medicine, UNSW Australia, Sydney, 2052, Australia.

[4]St. Vincent's Institute of Medical Research, Fitzroy, VIC 3065, Australia.

*Address correspondence to: Professor Herbert Herzog
Garvan Institute of Medical Research
384 Victoria Street, Darlinghurst NSW 2010, Australia
Email: h.herzog@garvan.org.au

Dr Kim Loh
St Vincent's Institute of Medical Research
9 Princes Street, Fitzroy VIC 3065, Australia
Email: kloh@svi.edu.au

SUPPLEMENTARY FIGURES

Supplementary Figure 1

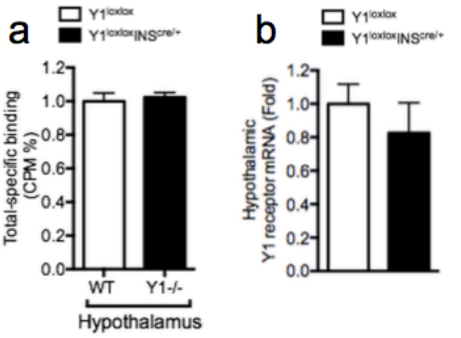

Supplementary Figure 1. Hypothalamic Y1 receptor expression in β-cell-specific Y1 receptor deficient mice. (**a**) Radioligand binding assays were performed on hypothalamic slices derived from $Y1^{lox/lox}$ and $Y1^{lox/lox}/INS2^{cre/+}$ mice using radiolabeled $^{Leu31,Pro34}NPY$ ligand. (**b**) Hypothalamic Y1 receptor expression from $Y1^{lox/lox}$ and $Y1^{lox/lox}/INS2^{cre/+}$ mice were determined by quantitative RT-PCR and RPL-19 was used as a housekeeping gene. Data are means ± SEM of 4-6 mice per group.

Supplementary Figure 2

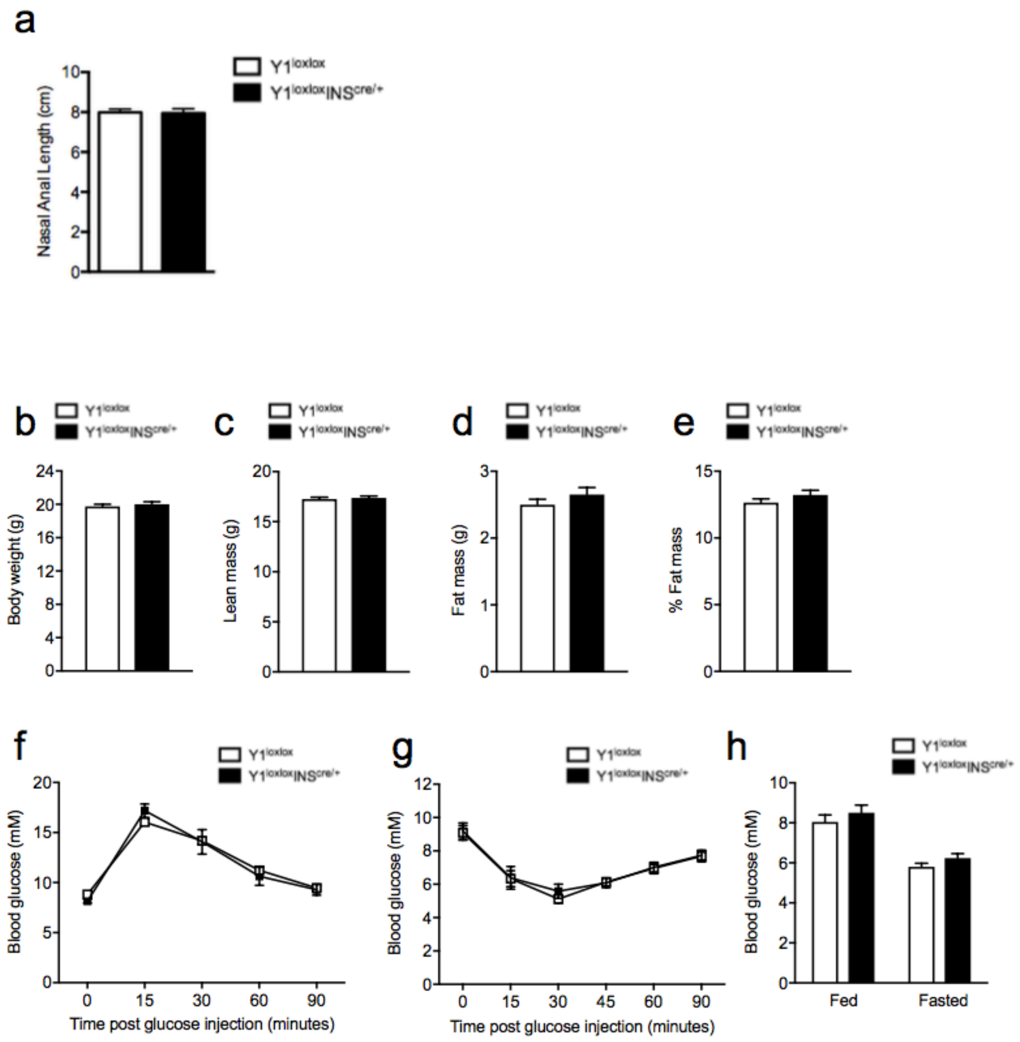

Supplementary Figure 2. Unaltered body length, body composition and glucose homeostasis in 7-8-week-old β-cell-specific Y1 receptor deficient mice. (**a**) Nasal anal length was measured in Y1$^{lox/lox}$ and Y1$^{lox/lox}$/INS2$^{cre/+}$ mice. (**b**) Body weight of 7-8-week-old Y1$^{lox/lox}$ and Y1$^{lox/lox}$/INS2$^{cre/+}$ mice. (**c-e**) Absolute and normalised body composition (lean and fat mass) determined by DXA scan for Y1$^{lox/lox}$ and Y1$^{lox/lox}$/INS2$^{cre/+}$ mice. (**f**) 7-8-week-old Y1$^{lox/lox}$ and Y1$^{lox/lox}$/INS2$^{cre/+}$ mice were fasted for 6h and glucose tolerance tests (1mg/kg) were performed. (**g**) 7-8-week-old Y1$^{lox/lox}$

and $Y1^{lox/lox}$/$INS2^{cre/+}$ mice were fasted for 4h and insulin tolerance tests (0.75mU/g) were performed. (**h**) Fasted and non-fasted blood glucose levels in 7-8-week-old $Y1^{lox/lox}$ and $Y1^{lox/lox}$/$INS2^{cre/+}$ mice were determined. Results are means ± SEM of 8-10 mice per group.

Supplementary Figure 3

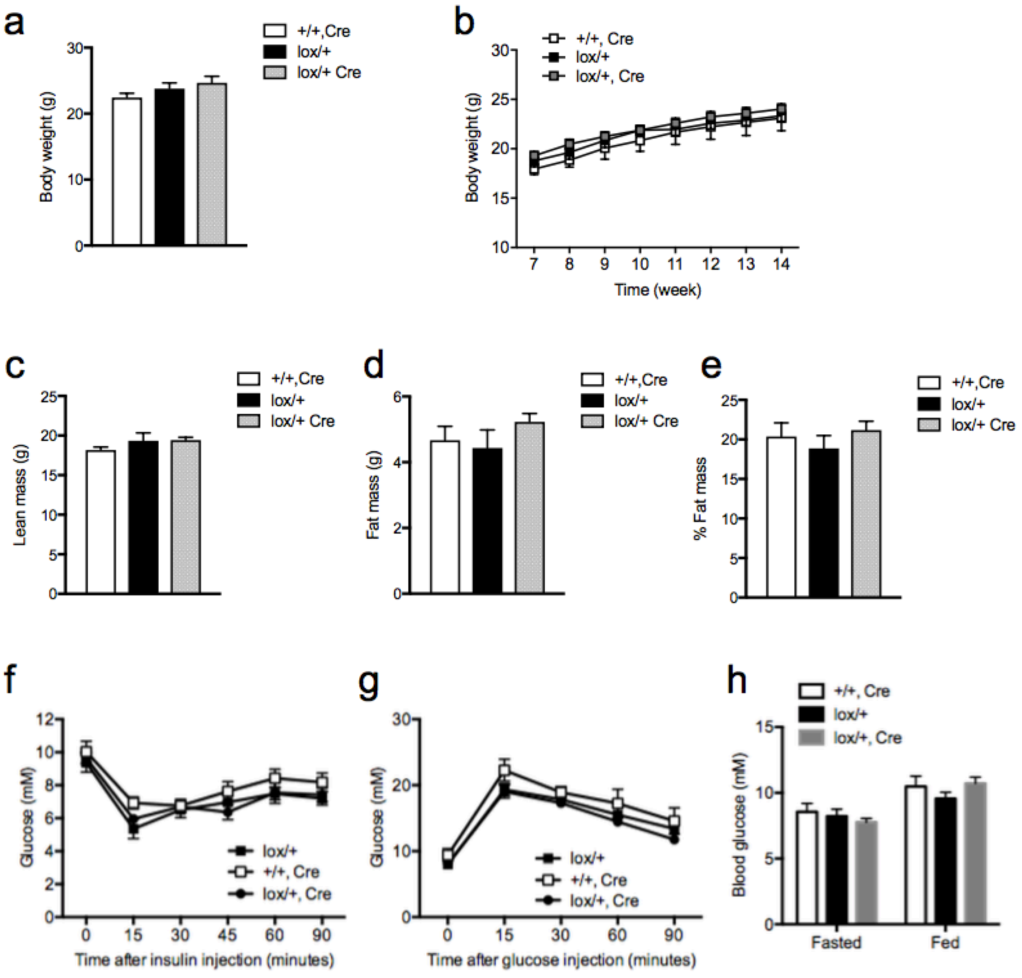

Supplementary Figure 3. Unaltered glucose homeostasis in INS2-Cre transgenic mice. (**a-b**) Body weight and body weight curve was determined in Y1$^{lox/+}$, Y1$^{lox/+}$/INS2$^{cre/+}$ and Y1$^{+/+}$/INS2$^{cre/+}$ mice. (**c-e**) Body composition (lean, fat mass and % fat mass) determined by DXA scan for 12-14-week-old Y1$^{lox/+}$, Y1$^{lox/+}$/INS2$^{cre/+}$ and Y1$^{+/+}$/INS2$^{cre/+}$ mice. (**f**) Chow fed 12-14-week-old Y1$^{lox/+}$, Y1$^{lox/+}$/INS2$^{cre/+}$ and Y1$^{+/+}$/INS2$^{cre/+}$ mice were fasted for 4h and insulin tolerance tests (0.75mU/g) were performed. (**g**) Chow fed 12-14-week-old Y1$^{lox/+}$, Y1$^{lox/+}$/INS2$^{cre/+}$ and Y1$^{+/+}$/INS2$^{cre/+}$

mice were fasted for 6h and glucose tolerance tests (1mg/kg) were performed. (**h**) Fasted and non-fasted blood glucose levels in $Y1^{lox/+}$, $Y1^{lox/+}/INS2^{cre/+}$ and $Y1^{+/+}/INS2^{cre/+}$ mice were determined. Data are means ± SEM of 4-8 mice per group.

Supplementary Figure 4

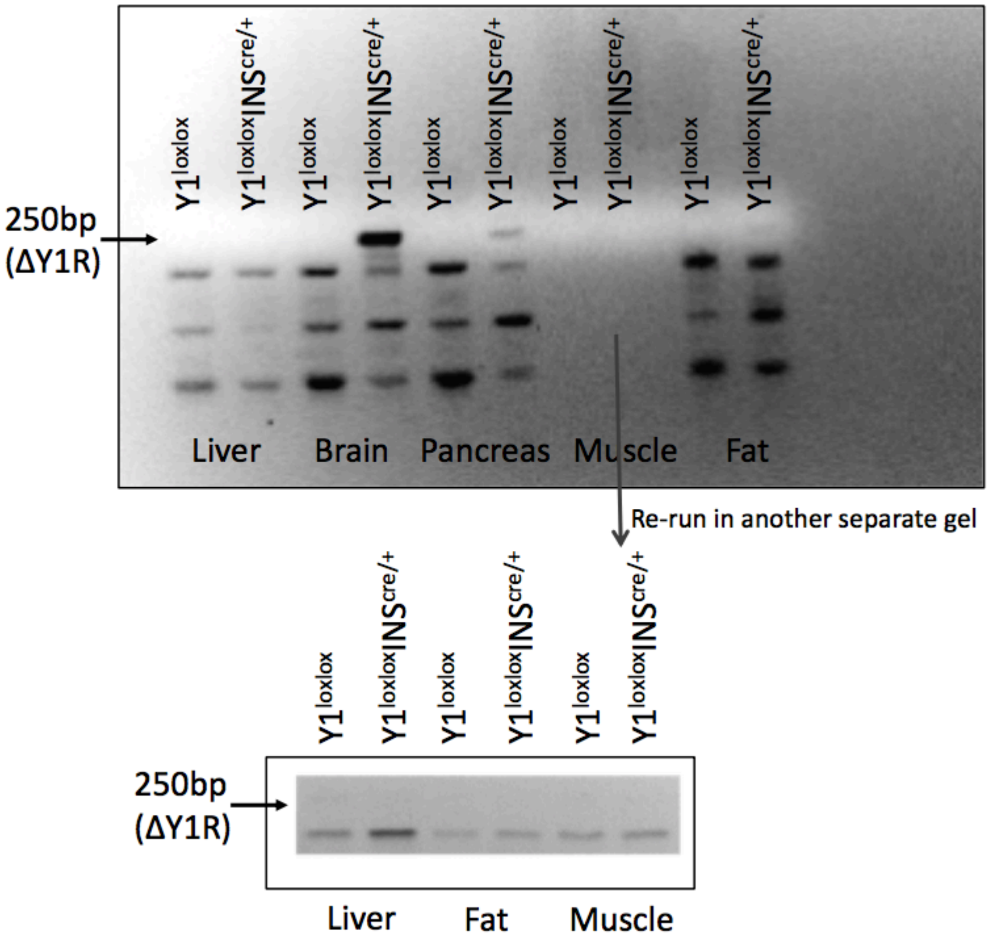

Supplementary Figure 4. Full length gels of figure 1B.

SUPPLEMENTAL EXPERIMENTAL PROCEDURES

Animal diets

All experimental mice were either fed on a standard chow diet (6% calories from fat, 21% calories from protein, 71% calories from carbohydrate, 2.6 kcal/g, Gordon's Specialty Stock Feeds, Australia) or a high fat diet (43% kilojoules from fat, 17% kilojoules from protein, 40% kilojoules from carbohydrate, 4.8 kcal/g, Specialty Feeds, Australia).

RNA extraction

To analyze gene expression in the brain and peripheral tissues, animals were killed by cervical dislocation followed by decapitation and tissue collection. All tissues were snap frozen in liquid nitrogen and stored at -80ºC for subsequent mRNA analysis as described below. Brains were collected and hypothalami were dissected and stored separately. A 5-cm segment of the intestine was collected from the gastro-duodenal junction, and the intestinal contents were removed by passing saline through the intestinal tube. White adipose tissue depots (right inguinal, right retroperitoneal and mesenteric), intrascapular brown adipose tissue, liver (the left hepatic lobe), testis, pancreas, femur and calvaria were collected. Whole tissues were homogenized in 2 ml TRI® reagent (Invitrogen, CA, USA). Chloroform (500 µl) was added and samples, which were vortexed for 15 seconds. Supernatant was collected after centrifugation at 12000 g for 10 minutes at 4ºC. An aliquot of the aqueous phase was mixed with an equal volume of isopropanol and incubated at room temperature for 15 minutes followed by centrifugation at 12000 g for 10 minutes at 4ºC. Supernatant was removed from the resulting RNA pellet, which was then rinsed with 75% ethanol in DEPC water.

Samples were centrifuged at 10000 g for 5 minutes at 4ºC. Supernatant was removed and the RNA pellet was air dried for 10 minutes and resuspended in 30 µl DEPC water that had been preheated to 55ºC. After overnight incubation at -80ºC, RNA quality and concentration was determined using the NanoDrop® ND-1000 Spectrophotometer (ThermoScientific, Wilmington, DE, USA).

Reverse transcription PCR

Synthesis of cDNA was performed using the QuantiTect Reverse Transcription kit (QIAGEN GmbH, Hilden, Germany). cDNA synthesis was preceded by elimination of genomic DNA by incubation of 1 µg of RNA in 12 µL total volume containing 2 µL of the gDNA Wipeout Buffer (QIAGEN GmbH, Hilden, Germany) at 42ºC for 2 minutes. A 6-µL master mix solution containing reverse transcriptase, oligo-dT, Mg2+ and dNTPs was then added to each RNA sample and reverse transcription was carried out at 42ºC for 15 minutes. The reaction was terminated by a 3-minute incubation at 95ºC.

Quantitative real-time PCR

Hypothalami, islets lysates, and peripheral tissues were collected and total RNA were isolated using Trizol Reagent (Sigma, St. Louis, MO) as per manufacturer's instructions. One microgram of total RNA was reverse transcribed into cDNA using Superscript III First-Strand Synthesis System (Invitrogen, Australia). Quantitative real-time PCR using primers for the Npy1r (Y1 receptor) gene (5'-CACAGGCTGTCTTACACG-3' and 5'-GCGAATGTATATCTTGAAGTAG-3'), Ribosomal protein L19 (Rpl-19) gene (5'-CTCGTTGCCGGAAAAACA-3' and 5'-TCATCCAGGTCACCTTCTCA-3'), Srebpf gene (5'-GGTTTTGAACGACATCGAAGA-3' and 5'-CGGGAAGTCACTGTCTTGGT-3') were performed on a Light-Cycler 480 Real-

Time PCR system (Roche, Switzerland) using SensiMix Probe (Bioline, Australia) following manufacturer's protocol. *Npy1r and* mRNA expression were normalized with a housekeeping gene, *Rpl-19*.

Analysis of body weight and composition

Body weight was determined once a week at the same time each week from 6 weeks of age onwards, unless otherwise stated. Mice were anesthesized by inhalation of 3% isoflurane and then scanned using dual energy X-ray absorptiometry (DXA) (Lunar Piximus II mouse densitometer; GE Healthcare, Chalfont St. Giles, Buckinghamshire, UK) to determine whole-body lean and fat masses as previously described (Baldock et al., 2009; Zhang et al., 2010).

Spontaneous and fasting-induced food intake studies

Measurements of spontaneous food intake in chow-fed mice were made at 14-16 weeks of age. For mice on the high fat diet, spontaneous food intake was measured at 16 weeks of age, after 8 weeks on a high fat diet, respectively. Mice were transferred from group housing on soft bedding to individual cages with a single paper towel in the bottom of the cage and allowed to acclimatize for at least 2 days. Mice were given *ad libitum* access to their assigned diet. Body weight, the weight of food in the hopper, the weight of food left on the cage floor and fecal output were recorded over four consecutive days. Food consumed was calculated as the weight of food taken from the hopper minus the weight of food spillage on the cage floor.

The effect of fasting on body weight and food intake was examined under normal chow or high fat diet. Food was removed from the cages for 24 hours, after which time mice

were given free access to their usual diet. Hopper weight, spillage, actual food intake and fecal output were recorded at 2, 4, 8 and 24 hours following re-feeding as described above. Body weight was recorded at all time points, including at 24 hours prior to re-feeding.

Indirect calorimetry

In order to determine energy expenditure, respiratory exchange ratio (RER) and physical activity, 14-16 weeks old mice fed on a normal chow diet, or 16-week old mice that had been maintained on the high fat diet for 8 weeks, were transferred to individual cages (20.1 x 10.1 x 12.7 cm) in an eight chamber open-circuit calorimeter (Oxymax Series; Columbus Instruments, Columbus, OH, USA). Temperature was maintained at 22°C, with an airflow of 0.6 L/min. Mice were acclimatized to the cages for 24 hours before beginning 24-hour recording of oxygen consumption (VO_2) and carbon dioxide production (VCO_2). RER was calculated as $VCO_2 \div VO_2$. Energy expenditure (kilocalories of heat produced) was calculated as calorific value (CV) x VO_2, where CV = 3.815 + 1.232 x RER as previously published (Melgar, 2007 #4915). Lean mass-normalized energy expenditure was calculated as energy expenditure divided by lean mass, which was determined immediately following the completion of indirect calorimetry using DXA as described above. Physical activity was also measured, using an OPTO-M3 sensor system (Columbus Instruments, Columbus, OH, USA), whereby ambulatory counts were a record of consecutive adjacent photo beam breaks in the horizontal space. Data for the 24-h monitoring period was presented as hourly averages for VO_2, VCO_2, RER, and energy expenditure as well as hourly summation for ambulatory activities. The calorimeter was calibrated before each use using highly pure primary gas standards (O_2 and CO_2).

Glucose and insulin tolerance tests

Food was removed from the hopper and mice were transferred to new cages with fresh bedding before 6 hours fasting. The injected glucose solution was prepared by diluting a sterile solution of 50% w/v glucose (Pharmalab, Lane Cove, NSW, Australia) to 10% using sterile physiological saline. Mice received i.p. administration of glucose at a dose of 1 mg/kg body weight in a volume of 10 µl/g body weight. Blood glucose levels were assessed using blood taken from the tip of the tail at 0, 15, 30, 60 and 90 minutes after glucose administration using the Accu-chek® Go glucometer (Roche, Dee Why, NSW, Australia). Blood samples were subsequently kept at room temperature before centrifugation at 13000 rpm for 2 minutes. Serum was collected and stored at -20°C for subsequent insulin assay as described below. For insulin tolerance tests, mice were fasted for 4 hours, administered with insulin (0.75 mU/g body weight) and blood glucose levels were assessed using blood taken from the tip of the tail at 0, 15, 30, 45, 60 and 90 minutes after insulin administration using the Accu-chek® Go glucometer (Roche, Dee Why, NSW, Australia).

Tissue collection

At 6-10 hours after onset of the light phase, chow-fed and high fat fed mice were culled by cervical dislocation followed by decapitation for collection of trunk blood. Glucose levels of trunk blood were assessed using the Accu-chek® Go glucometer (Roche, Dee Why, NSW, Australia). Trunk blood was allowed to clot at room temperature, centrifuged and the resulting serum was collected and stored at –20°C for subsequent hormone analyses as described below. White adipose tissue depots (right inguinal, right epididymal, right retroperitoneal and mesenteric), intrascapular brown adipose tissue

and liver were excised and weighed. The left hepatic lobe was collected and frozen in liquid nitrogen and stored at -80°C for subsequent lipid quantification as described below.

Oil Red O staining

To measure hepatic lipid infiltration, frozen livers were mounted in embedding medium (Tissue-Tek OCT compound, Sakura Finetek USA, Torrance, CA, USA) and cryostat sectioned at 6 µm and thaw-mounted on charged slides (SuperFrost® Plus, Menzel-Glaser, Braunschweig, Germany). Sections were fixed for 30 minutes in 4% PFA at 4°C, washed in 3 changes of 50% isopropanol, and then stained for 10 minutes with a solution of 1.2% Oil Red O in 60% isopropanol. The solution had been previously filtered on a 0.45 µm filter (MILLEX®-HA, Millipore, Carrigtwohill, Ireland). To quantify lipid infiltration in the liver, Oil Red O stained sections were assessed on a grey scale for stained pixels within a defined frame using a light microscope (Leica, Heerbrugg, Switzerland) at 40x magnification.

Radioligand-binding assay

Radioligand-binding studies were carried out on hypothalamic slices from WT and $Y1^{lox/lox}/INS2^{cre/+}$ mice using the Y1 preferring radiolabeled ligand, ^{Leu31,Pro34}NPY. Hypothalamic block were isolated and homogenize in homogenization buffer (10mM NaCl, 5mM MgCl2, 2.5mM CaCl2, 50mM Tris-HCl and 1mg/ml bacitracin) and subsequently incubated with ^{Leu31,Pro34}NPY for 2 hours. Horse serum were added and centrifuged to separate bound and free ^{Leu31,Pro34}NPY. Radioactivity was determined using a gamma-counter (Gamma Scint 4).

Statistical analysis

Genotype distribution in litters resulting from the breeding pairs were compared to the expected Mendelian ratio using a Chi-squared test. Litter sizes, percentage of male offspring and mortality rates in pups born to wild-type, homozygous knockout and heterozygous breeding pairs were analysed using one-way ANOVA.
